# The Importance of the “Time Factor” for the Evaluation of Inhibition Mechanisms: The Case of Selected HDAC6 Inhibitors

**DOI:** 10.3390/biology12081049

**Published:** 2023-07-26

**Authors:** Edoardo Cellupica, Gianluca Caprini, Gianluca Fossati, Doris Mirdita, Paola Cordella, Mattia Marchini, Ilaria Rocchio, Giovanni Sandrone, Andrea Stevenazzi, Barbara Vergani, Christian Steinkühler, Maria Antonietta Vanoni

**Affiliations:** 1 Research and Development, Italfarmaco Group, Via dei Lavoratori 54, 20092 Cinisello Balsamo, Italy; e.cellupica@italfarmacogroup.com (E.C.); g.caprini@italfarmacogroup.com (G.C.); g.fossati@italfarmacogroup.com (G.F.); p.cordella@italfarmacogroup.com (P.C.); m.marchini@italfarmacogroup.com (M.M.); i.rocchio@italfarmacogroup.com (I.R.); g.sandrone@italfarmacogroup.com (G.S.); a.stevenazzi@italfarmacogroup.com (A.S.); b.vergani@italfarmacogroup.com (B.V.); 2 Department of Biosciences, University of Milan, Via Celoria 26, 20133 Milano, Italy; doris.mirdita@unimi.it

**Keywords:** histone deacetylase, histone deacetylase 6, inhibition, kinetics, medicinal chemistry, hydroxamates, difluoromethyloxadiazoles, cancer, neurodegeneration, immunomodulation

## Abstract

**Simple Summary:**

Protein lysine acetylation is, with phosphorylation, the most common regulatory post-translational modification of proteins. Histone deacetylases (HDAC) catalyze the removal of acetyl groups from histone and non-histone proteins, participating in the modulation of several pathways. Histone deacetylase 6 is perhaps the most complex among histone deacetylases, comprising two catalytic domains, an *N*-terminal microtubule-binding domain and a C-terminal ubiquitin–binding domain. Interfering with its catalytic activity by using small synthetic molecules has been shown to be beneficial in the treatment of cancer, and neurological and immunological disorders. Thus, the development of potent and selective inhibitors of HDAC6 is an active field of medicinal chemistry. We shall here discuss the importance of monitoring the kinetics of onset and relief of inhibition to contribute important information on inhibition mechanisms during drug design/development campaigns using selected HDAC6 inhibitors as examples.

**Abstract:**

Histone deacetylases (HDACs) participate with histone acetyltransferases in the modulation of the biological activity of a broad array of proteins, besides histones. Histone deacetylase 6 is unique among HDAC as it contains two catalytic domains, an *N*-terminal microtubule binding region and a C-terminal ubiquitin binding domain. Most of its known biological roles are related to its protein lysine deacetylase activity in the cytoplasm. The design of specific inhibitors is the focus of a large number of medicinal chemistry programs in the academy and industry because lowering HDAC6 activity has been demonstrated to be beneficial for the treatment of several diseases, including cancer, and neurological and immunological disorders. Here, we show how re-evaluation of the mechanism of action of selected HDAC6 inhibitors, by monitoring the time-dependence of the onset and relief of the inhibition, revealed instances of slow-binding/slow-release inhibition. The same approach, in conjunction with X-ray crystallography, in silico modeling and mass spectrometry, helped to propose a model of inhibition of HDAC6 by a novel difluoromethyloxadiazole-based compound that was found to be a slow-binding substrate analog of HDAC6, giving rise to a tightly bound, long-lived inhibitory derivative.

## 1. Introduction

Post-translational modifications (PTMs) play important regulatory roles in cells [1]. Among them, acetylation of lysine residues is among the most abundant PTM after phosphorylation. PTMs depend on “writers” and “erasers”, as well as “readers”. In the case of acetylation, the modifications are introduced by histone acetyltransferases (HATs) and removed by histone deacetylases (HDACs). These enzymes were first discovered as histone modifiers in the nucleus, with acetylation typically promoting gene expression [2]. However, the number of non-histone and non-nuclear proteins undergoing acetylation/deacetylation is growing ([3,4] and references therein). Indeed, the biological processes other than gene expression, which are controlled by the acetylation state of some of their protein components, include cell trafficking, growth, division, migration and differentiation, and cell–cell contacts. Thus, acetyltransferases and deacetylases should probably be better defined as (protein) lysine acetyltransferases (KATs) and deacetylases (KDACs), respectively.

Controlling the lysine acetylation state of cellular proteins, by modulating the activities of acetyltransferases and deacetylases, has been shown to be beneficial for treating a broad array of diseases besides cancer [5].

HDACs have been classified into four classes based on sequence similarities. Class I, II and IV are zinc-dependent enzymes, while class III comprises sirtuins that are structurally unrelated to the zinc-dependent forms and use NAD^+^ as the coenzyme [6]. The number and type of enzymes endowed with lysine deacetylase activities is likely to increase as shown, for example, by the recent discovery that NDST3, which was previously known to catalyze *N*-deacetylation and *N*-sulfation of *N*-acetylglucosamine residues in heparan sulfate, is able to deacetylate microtubules lysine residues. Through such activity, NDST3 controls the microtubule-dependent assembly of the V-ATPase on the lysosomal membrane, which in turn controls lysosome acidification. Since lysosomes are key players in autophagy and are, therefore, implicated in several disorders, NDST3 may be a novel pharmacological target as other HDAC family members [7,8]. In support of its activity as a lysine deacetylase is its sensitivity to HDAC inhibitors, in particular those targeted to HDAC6.

HDAC6 belongs to class IIb of HDAC. It is unique among known HDACs, being characterized by the presence of two structurally related tandem catalytic domains [6,9,10,11]. Although it can deacetylate histones in vitro [12], it is mainly located in the cytoplasm. Among its protein substrates, acetylated tubulin was the first to be discovered [13]. The domain *N*-terminal to the first catalytic domain (CD1) has been recently found to function as a microtubule-binding domain besides harboring both a nuclear localization and a nuclear export signal sequence [14]. The region C-terminal to the second catalytic domain (CD2) is a ubiquitin-binding site characterized by a zinc finger. The domains preceding and following the catalytic domains are not essential for the enzymatic activity of HDAC6. However, the intrinsically disordered N-terminal microtubule-binding domain promotes deacetylation of tubulin. Thus, it modulates microtubule assembly by promoting protein–protein interaction [14]. Furthermore, it is important for HDAC6 dimerization, which, however, does not seem to affect the enzyme activity [15]. The C-terminal domain confers to HDAC6 a biological role unrelated to the deacetylase activity, by directing misfolded aggregated proteins to aggresomes through interaction with unanchored ubiquitin released by ataxin-3 deubiquitinase [16].

There is consensus on the fact that both HDAC6 catalytic domains can deacetylate histones [12], and that tubulin deacetylation is catalyzed by CD2 [13]. In particular, it has been recently shown that HDAC6 has a preference for acetylated tubulin dimers over microtubules [17]. Whether both CD1 and CD2 are catalytically active, or CD1 is just required to stabilize and/or to activate CD2 is still, to a large extent, an open question [18,19]. Recently, it has been proposed that CD1 preferentially deacetylates peptides with the acetylated lysine located at the C-terminal position, while CD2 prefers peptides with internally located acetyl-lysines [9,20]. Most synthetic substrates, which are routinely used to assay HDAC6 activity, appear to be recognized by CD2, as shown by studies on the isolated domains and/or on HDAC6 forms in which each one of the domains has been selectively inactivated by substitution of key catalytic residues (namely, His216 to inactivate CD1 and His611 to inactivate CD2) [10,19].

HDAC6, like other HDACs, is a promising pharmacological target also in the context of efforts to combat diseases other than cancer, especially those associated with inflammation [5]. Among HDAC inhibitors, vorinostat (SAHA, suberoylanilide hydroxamic acid) was the first molecule to receive approval from the Food and Drug Administration (FDA) for the treatment of cutaneous T-cell lymphoma, and its effect seems to be related to the activation of the expression of genes that eventually lead to apoptosis of malignant cells. However, like other pan-HDAC inhibitors, SAHA exerts a strong anti-inflammatory effect [21] the molecular mechanisms of which still need to be fully elucidated. Givinostat (also known as ITF2357) has been shown to be effective in ameliorating Duchenne muscular dystrophy in preclinical models, likely through its immunomodulatory role [22,23,24]. Results of a phase III clinical trial have been recently made available (www.Clinicaltrials.gov (accessed on 30 April 2023), clinical trial identifier: NCT02851797). More recently, it has even been proposed that treatment with HDAC inhibitors, especially HDAC6-specific ones, can help in the treatment of severe COVID-19, which is associated with a deregulated immune response [25].

A limitation to the use of the currently FDA-approved pan-HDAC inhibitors in the clinic [vorinostat (SAHA), romidepsin (FK228), belinostat and panobinostat] is their toxicity associated with the most effective dose [26,27]. Due to the non-overlapping roles of HDAC isoforms, finding isozyme-specific HDAC inhibitors would lead to a major breakthrough in the field, increasing the effectiveness of the compounds and lowering side effects.

Most inhibitors directed towards zinc-dependent HDAC have a common tripartite scaffold: a zinc-binding group targeting the catalytically essential zinc ion at the bottom of the active site cavity, a spacer and a cap group [28,29]. The spacer somehow mimics the acetylated lysine side chain of the protein substrate, and exploits specific interactions with the enzyme side chains decorating the active site leading from the enzyme surface to the bottom of the active site where the zinc ion is located. The cap group should also contribute to specificity by interacting with the enzyme surface surrounding the active site opening. The cap group has also been proposed to be amenable to modifications that could lead to the production of multifunctional inhibitors that could target both HDAC and a second target, as an alternative to multi-drug therapy [30].

For the assessment of the potency of inhibitors in medicinal chemistry programs, the concentration of the inhibitor causing a 50% decrease in activity of the target enzyme (IC_50_) in steady-state kinetic studies is typically determined. This is very often achieved by measuring the initial velocity of the reaction at a fixed time-point in the absence (v_o_) and presence (v_i_) of a series of inhibitors’ concentrations that are pre-incubated with the enzyme for a fixed time, prior to addition of the substrate to initiate the reaction. Such experimental design implies that the equilibrium between enzyme, inhibitor and substrate is rapidly established. Thus, the residual activity measured in the presence of a fixed (sub-saturating) substrate (S) concentration and varying inhibitor (I) concentrations will reflect the rapid partitioning among free enzyme (E) and its complexes with the inhibitor (EI) and other enzyme forms (namely, the enzyme–substrate complex (ES) for a simple reaction and a competitive inhibitor). Any deviation from a hyperbolic dependence of residual activity (v_i_/v_o_) upon inhibitor concentration, expressed by values of the “slope factor” (s) in Equation (1), will be typically interpreted as a sign of cooperative binding of the inhibitor to the enzyme and/or the formation of complexes between the enzyme and more than one inhibitor molecule.

(1) $$\frac{v_{i}}{v_{o}}\times 100={\left(\frac{100}{1+\frac{I}{{IC}_{50}}}\right)}^{s}$$

As discussed in several classic textbooks and papers (see e.g., [31,32,33,34,35]), monitoring the time-course of the reaction under various conditions, although labor intensive, should be preferred over fixed-time assays. Indeed, it may reveal cases of slow-binding and/or slow-release inhibitors that would be otherwise missed, leading to misleading results in terms of inhibitor potency, stoichiometry and mechanism of action (see Supplementary Figure S1). Examples of the importance of monitoring the kinetics of onset and release of inhibition will be provided below as emerging from studies on HDAC6.

Slow binding may be related to the low concentrations of inhibitors that one may need to use in the case of dissociation constants (K_i_) well below the nM range. In such studies, the concentration of the inhibitor needs to be of the same order of magnitude of the K_i_ value. Thus, the velocity of formation of the enzyme–inhibitor complex may be very low.

For example, for a K_i_ in the nM range, the apparent first-order rate constant of enzyme–inhibitor complex formation when the inhibitor is present at 1 nM (10^−9^ M), assuming that the process is limited by diffusion (10^9^ M^−1^ s^−1^), is 10^−9^ M × 10^9^ M^−1^ s^−1^ = 1 s^−1^, corresponding to a half-time of 0.693 s. Since, in steady-state assays, the time taken to mix the enzyme with substrate and inhibitor is at least 10-30 s, the equilibrium between enzyme, substrate and inhibitor is established when observation of the reaction starts. However, if the inhibition constant is in the pM (10^−12^ M) or fM (10^−15^ M) range, and the inhibitor needs to be used in those concentration ranges for activity measurements, the observed first-order rate constant for complex formation will fall in the range of 10^−3^ s^−1^ and 10^−6^ s^−1^. By taking into account transit times, it would take 10^3^ or 10^6^ s to form the enzyme–inhibitor complex. Thus, one would initially observe the velocity of the uninhibited reaction and a time-dependent decrease in the rate until a steady-state velocity is reached.

In other instances, the formation of the initial enzyme–inhibitor complex may be actually slow, or a slow conformational change needs to take place after formation of the initial enzyme–inhibitor complex in order to obtain the tight inhibited complex. Slow release of the inhibitor from such a tight complex leads to a long residence time of the inhibitor in the enzyme active site, which is a property that contributes to an actual drug’s efficacy [32,36,37,38].

In practical terms, when fixed-time assays are used for inhibitor screening, these types of very effective compounds would be missed, or complex dependencies of the percentage inhibition from the inhibitor concentration would be observed (see examples in Supplementary Figure S1). Indeed, when the onset of inhibition is slow, one needs to monitor the reaction for long times under conditions in which the velocity of the reaction in the absence of inhibitor (v_o_) remains constant. Under such conditions, one is able to determine the reaction velocity at the steady-state (v_s_), when the inhibitor is bound to the enzyme at equilibrium, and the apparent rate constant (k_obs_) for the onset of inhibition through the Morrison equation (Equation (2)). The latter quantifies product formation (P) during the time-course of the reaction, by taking into account how the reaction velocity changes over time (Equation (3)) ([39,40]; see also [31] for a comprehensive manual).

(2) $$\left[P]=v_{s}\times t+\frac{v_{i}-v_{s}}{k_{obs}}(1-e^{-k_{obs}t}\right)$$

(3) $$v=v_{s}+\left(v_{o}-v_{s}\right)\times e^{-k_{obs}t}$$

The dependence of k_obs_ from the inhibitor concentration will be linear for simple slow binding of the inhibitor of the type E + I → EI. However, it will be hyperbolic if the slow inhibition depends on a slow conformational change following the initial enzyme–inhibitor complex formation of the type E + I ↔ EI → (EI) *.

The nature of the conformational change may be difficult to identify. Furthermore, observing a linear relation between k_obs_ and inhibitor concentration may indicate a two-step reaction with a large dissociation constant of the initial enzyme–inhibitor complex. In any case, determining the IC_50_, the mechanism of inhibition and the actual K_i_ requires the use of the v_s_ values obtained under various conditions.

Low inhibition constants may also depend on slow release of the inhibitor from the enzyme active site, leading to a long residence time. The latter is a desired property of drugs as it guarantees that the target enzyme will remain inhibited for a significant fraction of time in the complex cell environment [32,36,37,38].

Slow release of the inhibitor from the enzyme active site can be detected by observing the time-course of product formation (or substrate consumption) when the substrate is added to the enzyme that has been pre-incubated with the inhibitor for a time long enough to establish equilibrium. In such a case, one observes a progressive increase in the reaction velocity, as the inhibitor is released from the active site, to reach a steady-state value (see [31]).

The process is also mathematically described by Equations (2) and (3). Under high substrate concentrations (and slow binding of the inhibitor), the observed rate constant that describes the transition from the initial (inhibited) reaction to the steady-state is the k_off_ of the inhibitor from the enzyme–inhibitor complex.

The same concepts apply also when one wishes to measure inhibitor binding or release by biophysical methods, in order to complement kinetic studies on the effect of the inhibitor on the enzyme activity (or when the target has no catalytic activity) [33,34]. Also in these cases, studying the kinetics of enzyme (protein)–ligand complex formation or dissociation is important. Low amounts of enzyme (protein) may prevent the use of complementary biophysical methods to study protein–ligand interaction. Also, when the quantity, solubility and stability of the protein (and/or of the inhibitor) are not a problem, methods based on changes of a parameter as a function of complex formation may be difficult to identify. Methods that monitor shifts in the mass of the species when the complex is formed may give signals too small to be interpreted when a small molecule binds to a large protein. Formation of the enzyme–inhibitor complex may not cause detectable changes in thermal stability of the enzyme, or in the absorbance or fluorescence of either the enzyme or the ligand, with respect to their complexes. Finally, observing protein–ligand complex formation does not necessarily imply that the enzyme activity of the complex is impaired. Thus, with enzymes as targets, monitoring the effect of ligands on their activity is key to inhibitor search and development.

Failure to recognize slow onset of inhibition will lead to missing the inhibitory effect of certain compounds, or to overestimate the IC_50_, and to observe deviations from a simple hyperbolic binding curve, as exemplified in Supplementary Figure S1. When the kinetic mechanism of inhibition is studied by varying the substrate concentration at different levels of inhibitors, double reciprocal plots will not give simple patterns consistent with competitive, non-competitive or uncompetitive models, again leading to complex interpretations and, in general, the potency of the inhibitor may be underestimated.

We shall, here, show how re-evaluating the kinetics of onset and relief of inhibition of HDAC6, by monitoring the time-dependence of the reaction in discontinuous assays, has contributed to clarify the mechanism of action of selected HDAC6 inhibitors, namely ITF2357 (Givinostat [41,42]) and ITF3756 ([43], Supplementary Figure S2) that are characterized by a hydroxamic zinc-binding group, and two inhibitors (Compound **1** and Compound **2**) characterized by a difluoromethyl-1,3,4-oxadiazole (DFMO) zinc-binding group. The former DFMO-bearing compound was recently studied in detail [44]. Compound **1** was shown to be a potent HDAC6 inhibitor with an unprecedented greater than 10^4^ selectivity for HDAC6, with respect to all other isoforms, including HDAC10 that also belongs to Class IIb HDAC. By combining kinetics, mass spectrometry, X-ray crystallography and modeling, Compound **1** was found to exhibit slow onset of inhibition due to a slow conformational change that follows the initial formation of the enzyme–inhibitor complex, but also to be a slow substrate of HDAC6 giving rise to a tightly bound inhibitory species, which evolves to yield stable products that establish rapid equilibrium with the enzyme and exhibit low affinity.

## 2. Materials and Methods

Reagents. HDAC6 Fluor-de-Lys Green substrate, the corresponding deacetylated standard and developing solution were from Enzo Life Sciences. Trichostatin A (TSA) was from Sigma-Aldrich. Inhibitors [Givinostat (ITF2357), ITF3756, Compound **1** and Compound **2**] were synthetized at Italfarmaco according to published procedures [43,44]. All other reagents were from Sigma-Aldrich.

Enzymes. Human HDAC6, as a chimeric form in which the full-length protein is preceded by glutathione S-transferase (GST), was purchased from BPS Bioscience.

Activity assays. The activity assay described here is based on the use of the commercially available Fluor de Lys Green substrate, which contains an acetylated lysine residue covalently bound through an amide bond to the amino group of a fluorophore, likely a rhodamine derivative. Deacetylation of the lysine residue makes the amide bond susceptible to trypsin cleavage with release of the fluorophore that is accompanied by fluorescence increase. The time-course of reactions was monitored as described in detail in the Supplementary Information section.

Data analysis. For activity assays in the absence of inhibitors, measured fluorescence values at different reaction times, after subtraction of the background fluorescence, were analyzed to determine the initial velocity of reactions from the initial linear part of the curve. In the presence of inhibitors, Equation (2) was used to determine the steady-state velocity and the observed rate of onset or relief of inhibition. If the onset and relief of inhibition were sufficiently fast, compared to the time of the assay, the steady-state velocity could be calculated from the linear part of the progress curve at long reaction times. The dependence of the k_obs_ of Equation (2) upon the inhibitor concentration was linear, with a non-zero intercept, in all cases tested. Thus, we could calculate apparent values of k_on_ (the second-order rate constant for formation of the enzyme–inhibitor complex) and k_off_ (the first-order rate constant for dissociation of the enzyme–inhibitor complex) for a simple E + I ↔ EI equilibrium from Equation (4). From the observed rates of complex formation and dissociation, an apparent dissociation constant of the complex could be calculated (Equation (5); [31,40]).

k_obs_ = k_on_^app^ [I] + k_off_ (4)

K_i_^app^ = k_off_/k_on_^app^ (5)

The steady-state velocities were used to estimate the IC_50_ and cooperativity (if any) from the “slope factor” (s) by calculating the percentage residual activity as a function of inhibitor concentration, and fitting to Equation (1).

The kinetic mechanism of the inhibition and the inhibition constant (K_i_) could be determined using the steady-state velocities of the inhibited reaction and Equation (6), which describes competitive inhibition, as established from visual inspection of double reciprocal plots and replots of slopes and intercepts.

(6) $$v=\frac{V\times S}{Km\times \left(1+\frac{I}{Ki}\right)+S}$$

In Equation (6), v is the steady-state velocity, V is the maximum velocity of the reaction, S is the substrate concentration, I is the inhibitor concentration, K_m_ is the Michaelis–Menten constant.

For inhibitor pairs that were found to establish equilibrium relatively rapidly with the enzyme, we could also determine whether they bind in a mutually exclusive way to the enzyme with a so-called “Yonetani-Theorell plot” [31,45]. In this analysis, the substrate concentration (S) is maintained constant at a sub-saturating concentration, and the concentration of one inhibitor (I) is varied in the presence of different levels of the second (J). The reaction velocity at a given I and J concentration pair (v_ij_) depends on the corresponding inhibition constants (K_I_ and K_J_) and an interaction factor **γ** (Equation (7)). If I and J bind to the same site on the enzyme in a mutually exclusive way, the plots of reciprocal velocity as a function of the varied inhibitor concentration, at different constant levels of the second, yield a set of parallel lines because the interaction factor **γ**→∞ (Equation (8)). In this case, the common slope (Equation (9)) depends on the velocity of the uninhibited reaction (v_o_) and the dissociation constant of the varying inhibitor (K_I_). The dependence of the intercepts on the concentration of the inhibitor kept constant at different levels (J) should be linear (Equation (10)). The initial velocity of the inhibited enzyme, and the inhibition constants K_I_ and K_J_ can be calculated.

(7) $$\frac{1}{v_{ij}}=\frac{1}{v_{o}}\times \left(\left(1+\right.\frac{\left[I\right]}{K_{I}}+\frac{\left[J\right]}{K_{J}}+\frac{\left[I\right][J]}{{\gamma K_{I}K}_{J}}\right)\;$$

(8) $$\frac{1}{v_{ij}}=\frac{1}{v_{o}}\times \frac{1}{K_{I}}\times [I]+\frac{1}{v_{o}}\left(1+\frac{\left[J\right]}{K_{J}}\right)$$

(9) $$slope=\frac{1}{v_{o}\times K_{I}}$$

(10) $$intercept=\frac{1}{v_{o}}+\frac{1}{v_{o}\times K_{J}}\times [J]$$

For data fitting the Grafit program (Erythacus Software Ltd., East Grinstead, UK) was used.

## 3. Results

### 3.1. Tested Hydroxamic and Non-Hydroxamic Compounds Are Slow-Binding and Slow-Release Inhibitors of HDAC6

**Figure 1:**
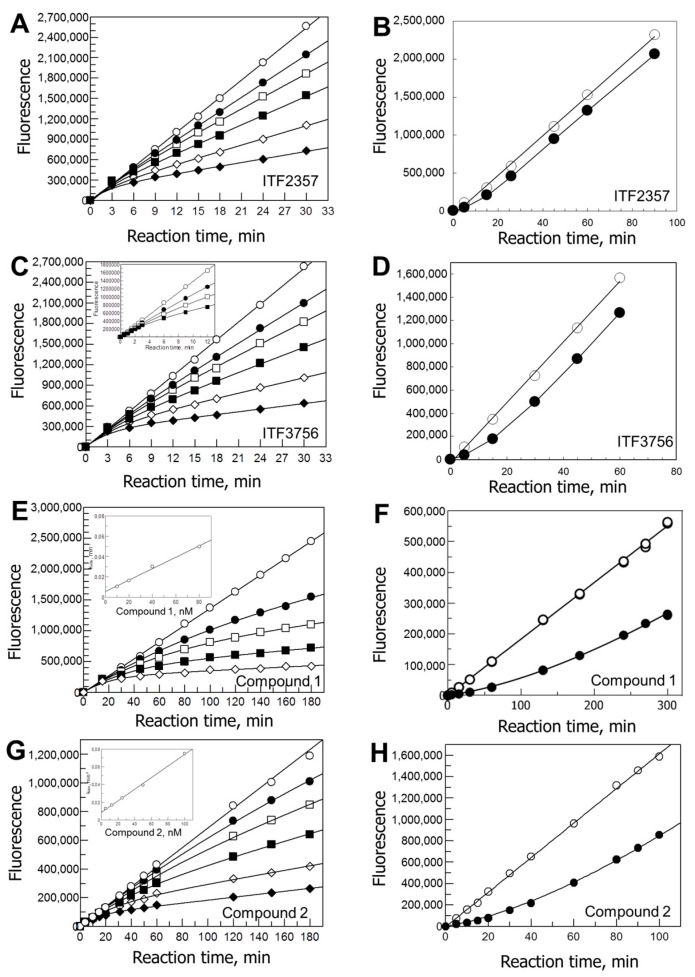
Kinetics of onset and relief of inhibition of HDAC6 by hydroxamic and non-hydroxamic compounds. Panels (**A**,**C**,**E**,**G**): kinetics of onset of inhibition. The reactions were started by the addition of GST-HDAC (typically 150 µL) to solutions (150 µL) containing varying inhibitors concentrations in 25 mM Tris/HCl pH 8, 130 mM NaCl, 0.05% Tween-20, 10% glycerol, 1% DMSO, and 4 μM Fluor de Lys Green. Aliquots of reactions were stopped at different times with TSA and subsequently developed, as described in detailed methods in Supplementary information. Panel (**A**): 0 (◯), 2 (●), 4 (□), 8 (■), 16 (⋄) and 32 (◆) nM ITF2357; 116 pg/μL GST-HDAC. Panel (**C**): 0 (◯), 1 (●), 2 (□), 4 (■) 8 (⋄) and 16 (◆) nM ITF3756; 116 pg/μL GST-HDAC; the inset shows a similar experiment done to study the early reaction times; here, withdrawn aliquots were added to the stop/developing solution. Panel (**E**): 0 (◯), 10 (●), 20 (□), 40 (■) and 80 (⋄) nM Compound **1**; 14.5 pg/μL GST-HDAC. Panel (**G**): 0 (◯), 5 (●), 12.5 (□), 25 (■), 50 (⋄) and 100 (◆) nM Compound **2**; 11.6 pg/μL GST-HDAC; reactions were terminated at different times by mixing with the stop/developing solution. The data were fitted to Equation (2) to determine the steady-state velocity values (v_s_), which were used to calculate IC_50_ and k_obs_ (Table 1). The initial velocity was set to the value measured in the absence of the inhibitor. The k_obs_ values showed in all cases a linear dependence upon inhibitor concentration (see insets of panels (**E**) and (**G**)). They were used to determine the mechanism of slow-binding inhibition, and estimates of k_on_, k_off_ and K_i_ (Table 1). Panels (**B**,**D**,**F**,**H**): kinetics of relief of inhibition in jump-dilution assays. GST-HDAC (2.9 ng/µL) was pre-incubated in the absence (◯) or presence (●) of ITF2357 (80 nM, Panel B) or ITF3756 (50 nM, Panel (**D**)) in a final volume of 10 µL in 25 mM Tris/HCl pH 8, 130 mM NaCl, 0.05% Tween-20, 10% glycerol, 0.5 mM TCEP, 1 mg/mL BSA and 0.3% DMSO). After 15 min at 25 °C, a solution (990 µL) containing 2 µM Fluor de Lys Green (approximately the K_m_ value) that had been equilibrated at 25 °C was added. Compound **1** (100 nM, Panel F) and Compound **2** (100 nM, Panel (**H**)) were pre-incubated with the enzyme (1.16 ng/µL, Panel F; 0.58 ng/µL, Panel H) for 120 min. A solution (990 µL) containing saturating Fluor de Lys Green (25 µM) was added to start the reaction. Reaction aliquots were withdrawn and stopped at the indicated times in the stop/developing solution. The data were fitted to Equation (2) to obtain estimates of the k_off_ value (Table 1). The parallel control (no inhibitor) samples demonstrated the stability of the enzyme during the experiment, and the linearity of the time-course of product formation during the reaction in the absence of inhibitor.

As shown in Figure 1 (left panels), with all tested HDAC6 inhibitors the progress curves of product formation were non-linear. In all cases, the initial reaction velocity was essentially independent from the presence of the inhibitor. At later times, the downward curvature of the plots, together with the linearity of product formation in the absence of the inhibitor, indicated slow onset of inhibition. For ITF2357 and ITF3756, a steady-state velocity was reached approximately 15 min after starting the reaction by addition of the enzyme (Figure 1A,C). Thus, the steady-state velocities could be measured by fitting the progress curves of product formation to Equation (2) or by interpolating the data points collected beyond 15 min with a straight line, obtaining similar values. The relatively fast onset of inhibition with ITF2357 and ITF3756 prevented us from accurately measuring k_obs_, and to determine the mechanism of slow onset of inhibition (whether a one-step process or a two-step one). For Compounds **1** and **2**, the onset of inhibition was sufficiently slow to allow us to determine k_obs_, which increased linearly as the inhibitor concentration increased, but with a non-zero intercept (Figure 1 E,G). Therefore, a model in which enzyme and inhibitor combine to yield the inactive enzyme–inhibitor complex in one step applies. From the slope of the lines and the intercepts (insets of Figure 1 E,G), one can calculate the apparent rate constants for formation of the inhibited complex and for its dissociation through Equation (4) (Table 1). Through Equation (5), the apparent dissociation constant of the EI complex could be calculated (Table 1). From jump-dilution assays of the type shown in Figure 1, right panels, the k_off_ was independently calculated through Equation (2) (or from the linear part of the curves) in the presence of saturating substrate, to minimize the possibility that the inhibitor rebinds to the enzyme after dissociation (Table 1). From the observed kinetics of onset and relief of inhibition, all inhibitors tested exhibited slow-binding and slow-release behavior.

### 3.2. Competitive Inhibition of HDAC6 by ITF3756 and Compound **2**

**Figure 2:**
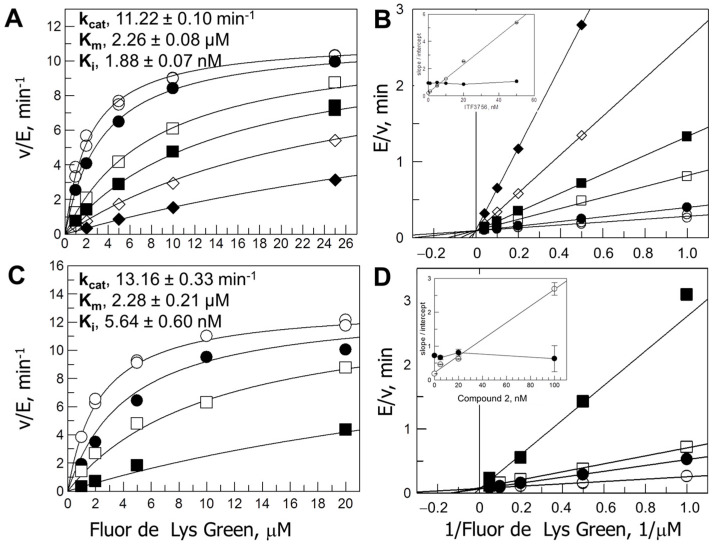
Steady-state kinetic analysis of the inhibition of HDAC6 by ITF3756 and Compound **2**. Panels (**A**,**B**). HDAC6 was added to solutions containing varying Fluor de Lys Green concentrations and constant levels of ITF3756 [0 (◯), 1 (●), 5 (□), 10 (■), 20 (⋄) and 50 nM (◆)]. Final assay composition was 25 mM Tris/HCl, pH 8, 130 mM NaCl, 0.05% Tween-20, 10% glycerol, 0.5 mM TCEP, 1 mg/mL BSA, 0.5% DMSO, varying substrate and inhibitor concentration. The time-course of the reaction was monitored by withdrawing aliquots at different times and adding them to an equal volume of the stop/developing solution as already described. The steady-state velocity values were calculated from the slope of the straight line interpolating the points in the linear part of the progress curves (20–60 min) and are expressed as v/E in min^−1^. v/E values were globally fitted to the equation for competitive inhibition (Equation (6)) (panel (**A**)) after inspection of double reciprocal plots (Panel (**B**)). The replots of the slope (open circles) and intercept (×10, closed circles) values obtained from independently fitting to straight lines; the data in the double reciprocal form shown in the inset of Panels (**B**–**D**) show the corresponding experiment with Compound **2** held at constant levels [ 0 (◯), 5 (●), 20 (□) and 100 nM (■)]. Here, the reactions were monitored for up to 3 h, and the steady-state velocities were calculated by fitting the progress curves to Equation (2).

The steady-state reaction velocities were measured in the presence of varying substrate concentrations and different levels of ITF3756 or Compound **2** by monitoring the time-course of the reactions. In both cases, the data were well-fitted by Equation (6), which describes competitive inhibition, as suggested by double reciprocal plots that gave a family of lines intercepting on the vertical axis the slopes of which exhibited a linear dependence on inhibitor concentration (Figure 2). As expected, the calculated K_i_ values were approximately half of the IC_50_ calculated at a substrate concentration corresponding to K_m_ (Table 1). Indeed, Equation (11) describes the relation between the IC_50_ and substrate concentration for competitive inhibition by taking into account K_m_ and K_i_ values. The relation can be derived by calculating the ratio between Equation (6) in the presence of an inhibitor concentration I, and the same equation in the absence of the inhibitor (I = 0). The ratio is set to 0.5, and the expression for the concentration of inhibitor that yields 50% residual activity (IC_50_) is calculated.

(11) $${IC}_{50}=\left(1+\frac{S}{K_{m}}\right)\times K_{i}$$

### 3.3. Mutual Exclusivity of Binding of ITF2357 and ITF3756

**Figure 3:**
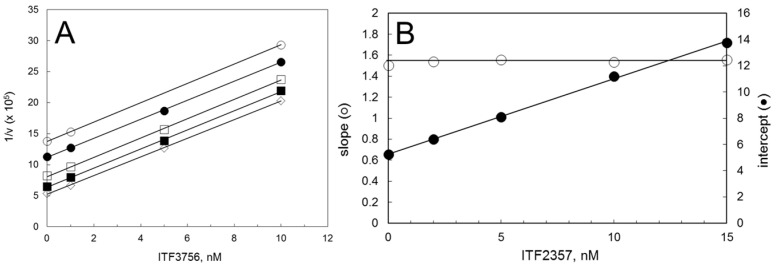
Yonetani–Theorell analysis of the binding of ITF2357 and ITF3756 to HDAC6. Panel (**A**): The reactions were started by adding aliquots of the enzyme solution (150 µL, 11.6 pg/μL final concentration) to an equal volume of solutions containing varying ITF3756 concentrations (0–10 nM, final concentrations) at fixed levels of ITF2357 [final concentrations: 0 (⋄), 2 (■), 5 (□), 10 (●) and 15 nM (◯)], and a fixed Fluor de Lys Green concentration (2 µM) in 25 mM Tris/HCl pH 8, 130 mM NaCl, 0.05% Tween-20, 10% glycerol, 0.5 mM TCEP, 1 mg/mL BSA, 0.5% DMSO. The time-course of reactions was monitored to calculate the steady-state velocity from the linear part of the curve. The reciprocal of the calculated velocity values (expressed as ΔF/min^−1^ × 10^5^) were plotted as a function of ITF3756 concentration. The series of assays was independently fitted with straight lines. Panel (**B**): Secondary plot of the slopes and intercept of the lines of Panel A as a function of ITF2357 concentration. The line through the slope values is the average value of the slopes (1.54 ± 0.02); the intercept values were fitted with a straight line of the type y = 0.57 * [ITF2357] + 5.27. v_o_, K_I_ and K_J_ values were calculated according to Equations (9) and (10) (Table 1).

Binding and release of ITF2357 and ITF3756 was sufficiently rapid (Figure 1) to allow us to test the mechanism of inhibition of ITF2357 once that of ITF3756 had been established (Figure 2). This could be accomplished through Yonetani–Theorell plots ([31,45], see data analysis section for details), which are useful for the characterization of series of inhibitors, once the mechanism of inhibition for one is known (in our case ITF3756, Figure 2). The approach is, in principle, valid under rapid equilibrium conditions in the absence of allosteric effects. However, for ITF2357 and ITF3756, equilibration with the enzyme was reached within a few minutes under all combinations of inhibitor concentrations. Thus, by monitoring the time-course of product formation of the assays, we could calculate and analyze the steady-state velocities’ values. Parallel lines were observed (Figure 3), indicating that, like ITF3756 that was demonstrated to be a competitive inhibitor of HDAC6 (Figure 2), ITF2357 is also a competitive inhibitor of HDAC6, with respect to the Fluor de Lys substrate. From the average of the slope values and the replots of intercepts (Equations (8) and (9)), the values of the inhibition constants K_I_ and K_J_ could be calculated as 3.4 nM (for ITF3756) and 9.2 nM (for ITF2357), respectively, which are in excellent agreement with the IC_50_ values also determined in the presence of 2 μM substrate concentration (Table 1).

## 4. Discussion

During studies aimed at identifying novel inhibitors of a target enzyme, including HDACs that are of increasing pharmacological interest, the potency of compounds is typically tested by measuring the residual activity in “fixed-time” assays, namely by determining the velocity of the enzyme-catalyzed reaction from product formed at a fixed reaction time, with or without pre-incubation of the enzyme with the inhibitor for a set time prior to substrate addition. Such an assay format is handy, and can even be automated in order to explore a large number of conditions (e.g., testing different inhibitors over broad concentration ranges, and comparison of the sensitivity of several enzyme isoforms, as in the case of the HDAC family of enzymes). However, the assay format implies that the reaction velocity is constant during the chosen interval of time in the absence and in the presence of varying inhibitor concentrations. This condition may not apply when the inhibitor binds slowly to the enzyme and/or it is slowly released from the enzyme–inhibitor complex. For slow-binding inhibitors, a pre-incubation of 10–30 min may not be sufficient to establish equilibrium. Thus, the calculated residual velocity may be overestimated leading to overestimating the IC_50_ value, and to deviations from simple binding, as highlighted by values of the slope factor (s) of Equation (1) being different from 1 (see Supplementary Figure S1). If the release of the inhibitor is slow, addition of substrate will cause slow redistribution of the enzyme among the free, and inhibitor- and substrate-bound forms. As a result, the residual velocity will be underestimated.

Although laborious, monitoring the time-course of product formation (or substrate consumption) in the presence of different inhibitor concentrations, over a sufficiently long time, will reveal cases of slow-binding and/or slow-release inhibition. Models describing the process can be drawn from analyses of how the initial and final velocities vary as a function of inhibitor concentrations, and from the inhibitor dependence of the apparent rate constant for the conversion of the initial (more) active species to the less active one. Reversibility of the inhibition can be checked with jump-dilution assays, which allow one to also determine the rate constant for the relief of inhibition. Monitoring the time-course of reactions in the absence of inhibitors is also a prerequisite for the set-up of robust assays. Indeed, it would reveal deviations from a linear increase in product concentration due to, e.g., lags or bursts prior to the onset of a steady-state, that would have to be taken into account in the modeling of the reaction.

Slow kinetics of binding and release of several types of inhibitors has been observed with class I HDACs, leading to thorough evaluation of their mechanism of action, potency and specificity [46,47,48,49].

In the case of HDAC6, the present study of the time-dependence of product formation during the reaction in the presence of inhibitors representative of the hydroxamic and non-hydroxamic class revealed that the onset of inhibition of HDAC6 is slow, regardless of the type of zinc-binding group of the compound (see also [44]). Slow binding is observed also with the pan-inhibitor TSA (Supplementary Figure S1), as previously reported for HDAC1-3 [47].

In all cases, the dependence of the rate constant observed for the onset of inhibition upon inhibitor concentration was linear, indicating a simple one-step mechanism of the type E + I ↔ EI in which the forward and reverse rate constants for complex formation and dissociation, respectively, are apparently slow. However, such linear dependence may be consistent with a two-step process in which the initial formation of the initial EI complex is characterized by a high dissociation constant. The EI complex would then slowly evolve to yield the tight EI* form. Thus, the kinetics of onset and relief of inhibition, and the associated conformational changes, appear to be worth investigating in greater detail with different inhibitor/HDAC couples, as part of studies of this interesting and important enzyme, and in the context of drug development.

To distinguish between the possible models explaining the experimental observations, and to establish the precise mechanism of onset of tight inhibition, several complementary methods may be needed.

**Figure 4:**
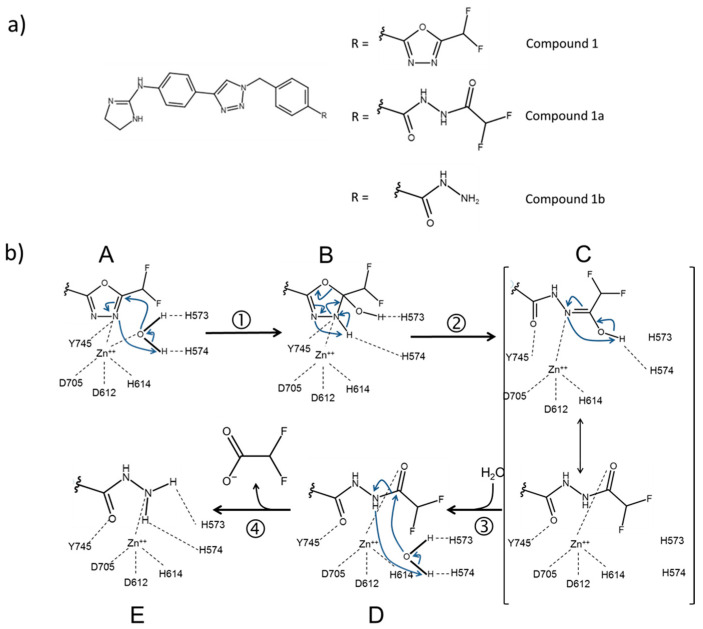
Proposed mechanism of hydrolysis of Compound **1** in HDAC6 active site. Panel (**a**) Chemical structures of Compound **1** and its stable ring opened (Compound **1a**) and hydrazide derivatives (Compound **1b**) deriving from two subsequent hydrolysis reactions. Panel (**b**) Schematic representation of: (**A**) Compound **1** bound to the active site of HDAC6 where a water molecule is held in place by H573, His574 and the Zn^++^ cation; (**B**) the unstable hydrated intermediate formed by attack of the water molecule (Step 1); (**C**) acyl-hydrazide intermediate (Compound **1a**) formed in step 2; (**D**) enzyme-acyl-hydrazide intermediate after the entrance of a second water molecule into the active site (step 3); (**E**) hydrazide (Compound **1b**) found in the zebrafish HDAC6 active site by crystallography (PDB code: 8A8Z) derived from the hydrolysis of Compound **1b** (step 4). Formulae in this and all other figures were drawn with Chemical Sketch Tool (https://www.rcsb.org/chemical-sketch).

This is exemplified by the study of the inhibition of HDAC6 by Compound **1**, which behaves as a slow-binding/slow-release inhibitor (Figure 1, Table 1). By combining information from kinetics, mass spectrometry, X-ray crystallography and *in silico* modeling, Cellupica et al., 2023 [44] showed that Compound **1** is actually a slow substrate of HDAC6 that is eventually hydrolyzed to the corresponding hydrazide and difluoroacetate (Figure 4). The hydrazide derivative was indeed found bound to the active site of the CD2 domain of zebrafish HDAC6 (zHDAC6-CD2) by X-ray crystallography. The latter enzyme was shown to be a valid model of human HDAC6 CD2 also with respect to the mode of onset and release of inhibition by Compound **1** and its derivatives. That the isolated enzymes in solution could convert Compound **1** into the hydrazide observed in the zHDAC6-CD2 active site was readily shown, ruling out a crystallographic artifact.

Computational work indicated that Compound **1**, bound to the HDAC6 CD2 active site, could be hydrolyzed in a series of consecutive reactions, which were experimentally tested by: (i) establishing the inhibition kinetics and mechanism of the postulated stable Compound **1** derivatives; (ii) identifying the compounds formed at different reaction times by mass spectrometry; and, also, (iii) by attempting to isolate and characterize complexes of the enzyme with the starting inhibitor and its postulated derivatives.

By monitoring the species released in solution in the presence of catalytic (although high) amounts of enzyme, HDAC6 indeed slowly converted the starting DFMO derivative (Compound **1**, Figure 4) into a first stable intermediate that could be identified as a ring opened acyl-hydrazide (Figure 4, Compound **1a**). The latter was then converted into the hydrazide observed in the zHDAC6-CD2 structure, and difluoroacetate (Figure 4, Compound **1b**). A mechanism of HDAC6-catalysed hydrolysis of Compound 1 was proposed and tested. Compound **1** can bind to free HDAC6 (A in Figure 4). An active site water molecule, held in place by the His573–His574 dyad and the zinc ion, would attack the sp^2^ electrophilic carbon adjacent to the difluoromethyl substituent, forming an unstable hydrated intermediate that cannot be isolated (Step 1 in Figure 4). The latter would evolve with ring opening and rearrangement to yield the stable acyl-hydrazide intermediate (Step 2 in Figure 4). A second water molecule could then hydrolyze the acyl-hydrazide intermediate to the hydrazide found in the crystal structure of HDAC6 and difluoroacetate (Steps 3 and 4 in Figure 4). Entrance of such a second water molecule may be hindered by the ligand, making this step very slow. The ring-opened acyl-hydrazide stable intermediate (Compound **1a** of Figure 4) and the final hydrazide product (Compound **1b**, Figure 4) were indeed synthetized. They were found to be weaker inhibitors of HDAC6 as compared with the starting Compound **1** (IC_50_ 1580 nM for the ring opened form, 329 nM for the hydrazide to be compared with 7.7 nM for Compound **1**).

Indeed, Compound **1** was converted into the acyl-hydrazide, and then into the hydrazide by HDAC6 in solution. Both Compound **1** derivatives were “fast-on” and “fast-off” inhibitors of HDAC6 that establish rapid equilibrium with the enzyme. Furthermore, under high enzyme concentration, only minute amounts of the acyl-hydrazide and hydrazide, but no Compound **1**, were found associated with the enzyme, indicating that the tightly bound inhibitor species is the unstable hydrated intermediate (or the stable ring-opened acyl-hydrazide intermediate, but only when bound in the active site). Interestingly, the second-order rate constant for formation of the acyl-hydrazide from Compound **1** by the zebrafish CD2 [(6.04 ± 0.22)×10^−3^ min^−1^] was similar to the observed rate constant for the release of Compound **1** from the enzyme in jump-dilution assays [(9.6 ± 1.1) × 10^−3^ min^−1^] [44]. This observation supported the concept that the acyl-hydrazide (or, more likely, the unstable hydrated intermediate) is the compound that is actually tightly bound to the enzyme, and that its formation may be the reason for the slow onset of inhibition by Compound **1**. However, no solvent deuterium kinetic isotope effects were observed on the kinetics of onset of inhibition ruling out that the slow onset of inhibition with Compound **1** is due to hydration (and ring opening) to yield the enzyme-bound hydrated unstable intermediate or the bound acyl-hydrazide. Rather, a conformational change of the initial (loose) complex between HDAC6 and Compound **1** is limiting the rate of the onset of inhibition. Interestingly, when Y745 was substituted with F in the zebrafish CD2 domain, inhibition by Compound **1**, and also hydrolysis of Compound **1** to the acyl-hydrazide, became fast. Thus, Y745, which is known to participate in binding the substrate carbonyl and in stabilizing the tetrahedral intermediate in the deacetylation reaction of the acetyl-lysine substrate, seems also to be involved in the conformational change that leads to inhibition, and in the stabilization of the tightly bound inhibitory intermediate. On the contrary, substitution of His574 with an alanyl residue led to loss of conversion of Compound **1** into its derivative, in agreement with the essential role of this residue as a general base in the physiological hydrolytic reaction, and with the fact that hydrolysis of Compound **1** is indeed enzyme-catalyzed. That HDAC6-selective inhibitors containing a DFMO moiety as the zinc-binding group are hydrolyzed by the enzyme, as described for the first time by Cellupica et al. [44], has been confirmed by Motlova et al. in a paper that was published while this manuscript was under review [50]. Compound **8** of Motlova et al. [50] shares with Compound **1** of Cellupica et al. [44] only the DFMO molecule. It was also found to undergo the two subsequent hydrolytic steps described for Compound **1** to yield the acyl-hydrazide and hydrazide species corresponding to Compound **1a** and **1b**, respectively, by both purified HDAC6 and by extracts from overexpressing cells. The quantum and molecular mechanics simulations reported in [50] nicely confirm and extend the proposed mechanism for Compound **1** hydrolysis [44].

## 5. Conclusions

Histone deacetylases are of increasing interest as therapeutic targets in several diseases [5]. Due to their non-redundant role in the cell, isoform-specific inhibitors are very much needed to abolish side effects and toxicity occurring with the use of pan-inhibitors. Different classes of HDAC inhibitors are being developed by combining different types of zinc-binding groups, linkers and cap moieties to anchor the compound into the active site, and exploit isoform-specific interactions with the residues lining the access to the active site and the enzyme surface to gain selectivity [28].

Here, we showed that monitoring the time-dependence of the onset and relief of the inhibition by selected compounds is a valuable approach to clarify their mode of binding to the enzyme and to rule out complex behaviors such as, for example, cooperativity effects. Our findings stress once more the importance of taking into account the kinetics of processes as part of their characterization. The example of Compound **1** also highlights the importance of combining enzyme kinetics with other approaches to actually make progress in the field of inhibitor development.

To our knowledge, among the several classes of HDAC inhibitors that have been co-crystallized with HDAC forms, only in the case of derivatives using the DFMO moiety as the zinc-binding group (Compound **1** of [44], this manuscript and Compound **8** of [50]), an enzyme-catalyzed irreversible modification of the ligand has been observed. Whether this may happen with other compounds bearing different types of chemical groups, and/or with HDACs of different classes, may be worth establishing by analyzing the species present in solution at varying incubation times with the enzyme.

As exemplified by the case of Compound **1**, should an enzyme-modified species be the actual tight inhibitor, one would be tempted to conclude that the observed slow rate of onset of inhibition corresponds to the enzyme-catalyzed conversion of the initial compound to generate the actual inhibitor in the enzyme active site. However, a conformational change preceding chemical modification of the ligand may be the cause of the slow onset of inhibition as demonstrated for Compound **1** [44].

Slow binding to HDAC6 has been observed with TSA, ITF2357 (Givinostat) and ITF3756, for which there is no evidence of enzyme-catalyzed chemical modification. Thus, slow onset of inhibition through a conformational change seems to be a property of the enzyme. However, with the current knowledge, whether a compound may act as a slow-binding/slow-release inhibitor cannot be predicted as the slow onset and relief of inhibition does not simply correlate with, for example, the type of zinc-binding group. Gathering information on the kinetics of binding and release of inhibitors of different structures to/from HDAC of different classes may eventually lead to shed light on this aspect that can be exploited for selective and potent drug design. As mentioned above, to our knowledge, slow inhibitor binding has been reported only for Class I HDAC members [46,47,48,49], and now for HDAC6, a class IIb member ([44], this work).

Several compounds based on fluoroalkyl oxadiazole zinc-binding groups are being developed as HDAC inhibitors [28,44,50,51,52], along with others that incorporate oxadiazole moieties in the linker and cap regions [53]. The finding that Compound **1** is a slow-binding inhibitor of the enzyme, due to a slow conformational change that precedes its hydration to yield a tightly bound species, characterized by a long residence time, may open new avenues for both the characterization of the mechanism of action of these compounds, and, hopefully, the development of a novel class of mechanism-based HDAC inhibitors. Indeed, slow binding has been recently observed, but not discussed, for HDAC6 in the presence of T-518 (Figure 1B of [51]), another fluoroalkyl oxadiazole-derivative. Interestingly, also HDAC3 (a class I representative) and HDAC9 (a class IIa HDAC) hydrolyze Compound **1** to yield the ring-opened hydrazide and the final hydrazide, although at a lower rate, and at higher inhibitor concentrations than those used for HDAC6 [44].

## Figures and Tables

**Table 1:** Summary of parameters describing the inhibitory effect of hydroxamic and non-hydroxamic HDAC6 inhibitors.

Inhibitor	IC_50_, nM (s) ^a^	K_i_^app^, nM ^b^	K_i_, nM ^c^	k_on_^app^, M^−1^ min^−1 b^	k_off_, min^−1 b^
ITF2357	8.0 ± 0.4 (1.0 ± 0.06)	ND 9.2 ^e^	-	ND	ND
ITF3756	3.40 ± 0.07 (1.04 ± 0.03)	ND 3.4 ^e^	1.9 ± 0.1	ND	ND
1	6.6 ± 0.34 (0.96 ± 0.04)	8.90 ± 0.23	ND	5.6 ± 0.03 × 10^5^	5.0 ± 0.13 × 10^−3^ (6.5 ± 0.2 × 10^−3^)^d^
2	10.8 ± 0.60 (0.81 ± 0.04)	13.0 ± 1.4	5.6 ± 0.6	6.5 ± 0.2 × 10^5^	8.5 ± 0.9 × 10^−3^ (11 ± 0.2 × 10^−3^)^d^

^a^ The data from experiments shown in Figure 1 were used to calculate the IC_50_ by calculating percentage residual activity from the steady-state velocities (v_s_) and the initial velocity measured in the absence of inhibitors, and fitting to Equation (1). The slope factor (s) is in parenthesis. ^b^ The apparent K_i_, k_on_ and k_off_ were calculated by fitting the rate constants for conversion into the inhibited complex at varying inhibitor concentrations (k_obs_) to Equations (4) and (5). ^c^ The K_i_ was calculated from Equation (6) that describes competitive inhibition using the steady-state velocity calculated from progress curves at varying substrate concentrations in the presence of varying inhibitor concentrations (Figure 2). ^d^ The value was determined from jump-dilution assays. For ITF2357 and ITF3756, the onset of inhibition (k_obs_) and inhibitor release from the enzyme were too fast to measure precisely. Thus, the values could not be determined (ND). ^e^ For ITF2357 a competitive mechanism of inhibition was determined with Yonetani –Theorell plots (Figure 3) knowing that ITF3756 competes with the substrate for binding to HDAC6 (Figure 2). The apparent inhibition constants of both ITF2357 and ITF3756 were determined at 2 µM Fluor de Lys Green, a concentration corresponding to the K_m_ value.

## Data Availability

Data are contained within the article, Supplementary material and cited work published elsewhere.

## Supplementary Materials

The following supporting information can be downloaded at https://www.mdpi.com/article/10.3390/biology12081049/s1: Figure S1: Supplementary Figure S1. Effect of the rate of onset of inhibition on the determination of IC_50_ values. Figure S2: Supplementary Figure S2. Structures of ITF2357 (Givinostat), ITF3756, Trichostatin A (TSA). Detailed activity assay methods.

## Abbreviations

BSA	bovine serum albumin
CD	catalytic domain
DMSO	dimethylsulfoxide
DFMO	difluoromethyloxadiazole
HAT	histone acetyl transferases
HDAC	histone deacetylase
IC_50_	inhibitor concentration that halves the reaction velocity
KAT	Lysine acetyltransferase
KDAC	Lysine deacetylase
NAD^+^	nicotinamide adenine dinucleotide (oxidized form)
PMT	post-translational modifications
TCEP	tris(2-carboxyethyl)phosphine
SAHA	suberoylanilide hydroxamic acid
TSA	Trichostatin A; [R-(E,E)]-7-[4-(Dimethylamino)phenyl]-*N*-hydroxy-4,6-dimethyl-7-oxo-2,4-heptadienamide
