# Supplementary material for: The Importance of the “Time Factor” for the Evaluation of Inhibition Mechanisms: The Case of Selected HDAC6 Inhibitors
Source: Biology (Basel). 2023 Jul 26;12(8):1049. doi: 10.3390/biology12081049 (PMC10452033; doi:10.3390/biology12081049)
Supplement: Supplementary file 1 [file biology-12-01049-s001.zip › biology-2449650-supplementary.pdf]

The importance of the "time factor" for the evaluation of inhibition mechanisms: the case of selected HDAC6 inhibitors.

Edoardo Cellupica [1], Gianluca Caprini [1], Gianluca Fossati [1], Doris Mirdita [2], Paola Cordella [1], Mattia Marchini [1], Ilaria Rocchio [1], Giovanni Sandrone [1], Andrea Stevenazzi [1], Barbara Vergani [1], Christian Steinkühler [1,*], and Maria Antonietta Vanoni [2,*]

[1] Research and Development, Italfarmaco Group, Via dei lavoratori 54, Cinisello Balsamo (Italy)
[2] Department of Biosciences, University of Milan, Via Celoria 26, Milano (Italy)
* Correspondence: maria.vanoni@unimi.it; c.steinkuhler@Italfarmacogroup.com

Supplementary information

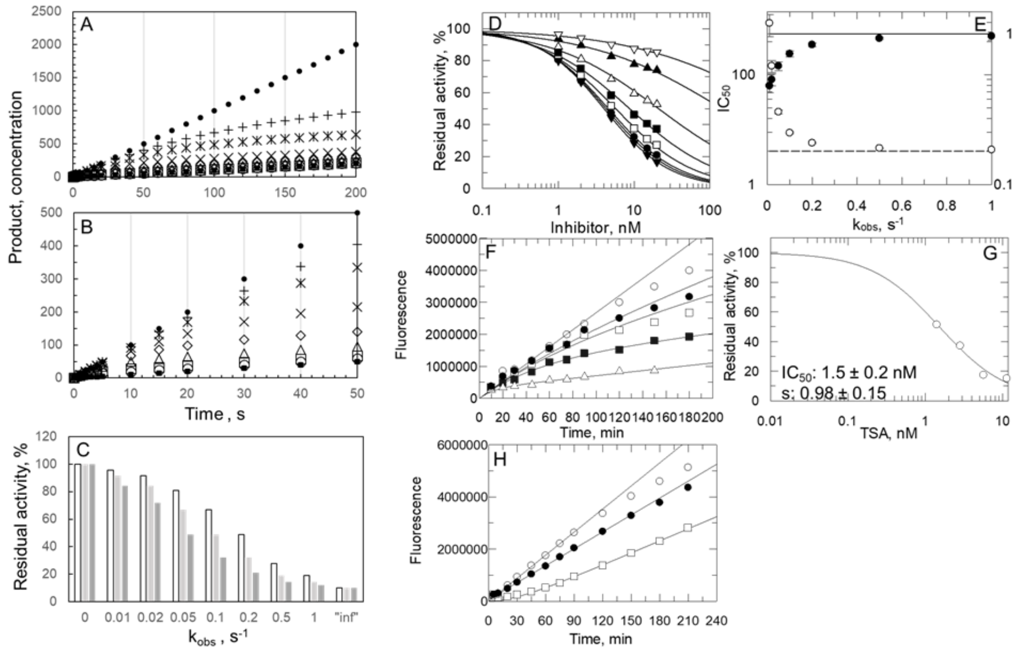

Supplementary Figure S1. Effect of the rate of onset of inhibition on the determination of IC_{50} values for an inhibitor competitive with respect to the varied substrate.

Panel A) Simulation of progress curves for varying k_{obs} [1 s^{-1} (open circles), 0.5 s^{-1} (open squares), 0.2 s^{-1} (open triangles) , 0.1 s^{-1} (open diamonds), 0.05 s^{-1} (crosses), 0.02 s^{-1}(stars) , 0.01 s^{-1} (plus sign)] at fixed initial velocity v_i (10) and steady-state velocity v_s (1). The units of velocity values will be chosen depending on the enzyme properties. The curves obtained in the absence of inhibitor (small closed circles) and in the presence of an inhibitor that establishes rapid equilibrium (small closed squares) are shown for comparison. Panel B) The curves of Panel A are shown on a more expanded scale to highlight differences at the early reaction times. Panel C) The percentage residual activity that would be calculated in fixed time assay formats in which the velocity of the reaction is calculated at 10 s (white bars), 20 s (light grey), 40 s (dark grey) is shown as a function of the k_{obs} . «0» indicates the velocity measured in the absence of inhibitor. «Inf» indicates the velocity when rapid equilibrium is established (k_{obs} tends to infinity). Panel D) Calculated residual velocity when the

velocity is measured at a reaction time of 40 s at different values of k_{obs} when the inhibitor concentration is varied between 0 and 20 nM, assuming the following parameters: K_i, 2 nM, K_m = 5 mM, S = 5 mM, V_{max} = 10 mM min^{-1} . The data calculated when rapid equilibrium is established are also shown. The curves are the best fit of the data to Eq 1 (main text). Panel E) Dependence of the values of IC_{50} and s (slope factor) and the associated errors when the data of panel D were fitted to Eq 1. Note that the IC_{50} (left axis) and slope factor values (right axis) are plotted on a logarithmic scale. The horizontal lines indicate the true slope factor of 1 and the expected value of IC_{50} (4 nM) taking into account the true value and that the substrate concentration was held constant at the K_m value. Panel F) Qualitative analysis of the rate of onset of inhibition of HDAC6 by the pan-inhibitor TSA as established by progress curves of product formation in reaction mixtures containing HDAC6, 25 μM Fluor-de-Lys Green substrate and 0 nm (open circles), 1.41 nM (closed circles), 2.83 nM (open squares), 5.66 nM (closed squares) and 11.32 nM (open triangles) TSA. The data in the absence of TSA were fitted with a straight line in the initial part of the curve to obtain the estimate of the initial velocity of the reaction. The curves are the fit of the data to Eq. 6. However, the experimental setting was not designed to accurately measure the observed rate constants for onset of inhibition especially at the low TSA concentrations where part of the curvature at the longer reaction times is also due to decrease of velocity due to substrate consumption. Panel G) The steady-state velocities estimated by fitting the data of panel F were fitted with Eq 1 to obtain estimates of the IC_{50} and the slope factor. Panel H) Qualitative analysis of the rate of TSA release from HDAC6 that had been pre-incubated with TSA (0 nM, open circle; 5 nM, closed circles; 10 nM , open squares) for 90 min prior to 100-fold dilution in the presence of 25 μM Fluor-de-Lys Green substrate. The curve describing the time-course of product production in the absence of TSA was fitted with a straight line in the 0-90 min range. The steady state velocity of the reaction in the presence of 0.05 nM TSA was also calculated by fitting the data from 30 min to a straight line. The time-course of product formation in the sample that had been preincubated with 10 nM TSA prior to 100-fold dilution (0.1 nM TSA in the assay) was fitted with Eq 6 obtaining an apparent k_{off} of 0.032 ± 0.008 min^{-1}

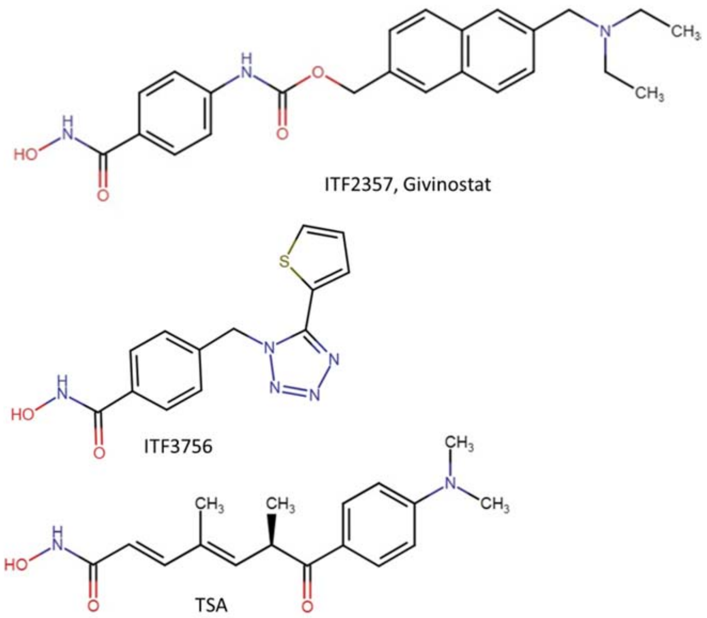

Supplementary Figure S2. Structures of ITF2357 (Givinostat), ITF3756, Trichostatin A (TSA). The structure of Compound1 is in Figure 4 (main text).

Detailed activity assay methods

The enzyme, which is stored in 5-10 μl aliquots at -80°C, is diluted to 2-6 ng/μl in ABTB [25 mM Tris/HCl buffer, pH 8, 130 mM NaCl, 0.05 % Tween-20, 10 % glycerol, 1 mM tris(2-carboxyethyl)phosphine (TCEP) and 2 mg/ml bovine serum albumin (BSA)] to prepare a "working solution", which is stable for at least 12 h when kept on ice. If needed, a more diluted solution in the same buffer (typically 0.6 ng/μl) is prepared prior to starting the assays in order to be able to add 1-50 μl aliquots to the reaction mixture. Substrate and inhibitor stock solutions (in DMSO) are diluted in assay buffer AB (25 mM Tris/HCl buffer, pH 8, 130 mM NaCl, 0.05 % Tween-20, 10 % glycerol).

To monitor the onset of inhibition, the assays are started by adding a small aliquot of concentrated enzyme to the assay mixture containing substrate and inhibitor that had been equilibrated at 25°C, or by mixing the enzyme solution in ABTB with an equal volume of a solution containing substrate and inhibitor in AB. The buffer composition is adjusted using volumes of AB and ABTB in order to carry out the reaction in 25 mM Tris/HCl, pH 8, 130 mM NaCl, 0.05 % Tween-20, 10 % glycerol, 1 mg/ml BSA, 0.5 mM TCEP. Depending on the substrate and inhibitor concentrations, DMSO is also included in the buffer in order to maintain its concentration constant. Control experiments showed that BSA at 1 mg/ml, TCEP at 0.5 mM optimize stability and activity of HDAC6, while DMSO up to 1 % does not affect stability and activity. Carrying out the reaction at 25°C (close to room temperature) instead of 30°C or 37°C minimizes variability due to (small) temperature changes during the assays and increases the stability of the enzymes during (long) reactions. Under these conditions, the K_M for Fluor de Lys Green was 2±0.2 μM and k_{cat} was 12 ± 0.2 min^{-1}.

To monitor the relief of inhibition in "jump-dilution assays", concentrated enzyme was pre-incubated with the inhibitor for a time long enough to ensure that the equilibrium between enzyme and inhibitor was established. The reaction was started by adding the substrate-containing buffer in order to rapidly dilute the enzyme and inhibitor containing solution 10-100-fold to reach an inhibitor concentration well below its K_i.

At different times after the start of the reaction, 60 μl aliquots are withdrawn and transferred to Eppendorf tubes containing an equal volume of Stop-Developing solution. The latter is prepared by a 200-fold dilution of the commercial Fluor de Lys Green Developer (Enzo Life Sciences) and addition of TSA (2 μM final concentration) in 50 mM Tris/HCl buffer, pH 8, 137 mM NaCl, 2.7 mM KCl, 1 mM $MgCl_2$ or by preparing a 0.4 mg/ml trypsin (from a 80 mg/ml stock in 1 mM HCl), 2 μM TSA solution (from a 2 mM stock in DMSO) in the same buffer. 25 min after the last withdrawal, after brief spinning at room temperature, 100 μl aliquots of each assay are transferred to wells of Half-Area plates (Perkin Elmer). The intensity of light emitted at 535 nm upon excitation with light at 485 nm is measured with an Ensight MultiMode plate reader (Perkin Elmer). The emission intensity at 600 nm was sometimes measured in order to distinguish between loss of linearity of the product formation due to enzyme inactivation or to signal saturation, and to monitor the reaction for longer times by delaying signal saturation. One or more parallel samples devoid of enzyme are also prepared and treated to obtain blank values to correct the measured fluorescence intensity values. As an alternative 30 μl of assay mixtures can be transferred to Eppendorf-type tubes containing 30 μl 16 μM TSA to stop the reaction. After withdrawal of the last sample, 60 μl of a 200-fold dilution of the commercial developing solution is added to each quenched sample. After 25 min, a 100 μl aliquot of each sample is transferred to the plate for fluorescence measurements.

In order to convert fluorescence intensity values into concentration of product formed, a calibration curve was built by diluting the Fluor de Lys Green deacetylated standard into the assay mixtures (60 μl, 0.4 – 50 nM) at 25°C, mixing with an equal volume of the Stop-Developing solutions and proceeding as described for the assays.

For jump-dilutions assays, 10 μl of a solution containing enzyme and inhibitor are incubated at 25°C for a given time. An aliquot of substrate solution (typically 990 μl), pre-equilibrated at 25°C, is added to dilute the inhibitor and to initiate the reaction. At different times, aliquots are withdrawn and handled as described. Parallel samples devoid of enzyme or inhibitor are also prepared and treated to obtain background fluorescence values and the time-course of the uninhibited reaction, respectively.
